# Synchronous primary intrapulmonary and mediastinal thymoma-A case report

**DOI:** 10.1186/1749-8090-5-69

**Published:** 2010-08-28

**Authors:** Zuoqing Song, Xiaohong Xu, Shujun Li, Sen Wei, Jun Chen, Qinghua Zhou

**Affiliations:** 1 Department of Lung Cancer Surgery, Tianjin Key Laboratory of Lung Cancer Metastasis and Tumor Microenvironment, Tianjin Lung Cancer Institute, Tianjin Medical University General Hospital, Tianjin 300052, China

## Abstract

We report an extremely rare case of Synchronous primary intrapulmonary and mediastinal thymoma in a Chinese patient. We describe the histological and radiological findings, which support the possibility of multicentric thymoma. Resection of the mass in the left anterior superior mediastinum and upper lobectomy of right lung were performed, with lymph Nodes clearance, superior vena cava, left and right brachiocephalic veins resection, reconstruction of left brachiocephalic vein to right auricle and reconstruction of right brachiocephalic vein to superior vena cava.

## Introduction

Thymomas are tumors derived from thymic epithelial cells and have an incidence of 0.15 per 100000[1]. Primary intrapulmonary thymomas are defined as thymomas arising in an intrapulmonary location without an associated mediastinal component and are very rare[2]. Here we present a successfully resected case of synchronous primary intrapulmonary and mediastinal thymoma with vascular reconstruction.

## Case report

**Figure 1:**
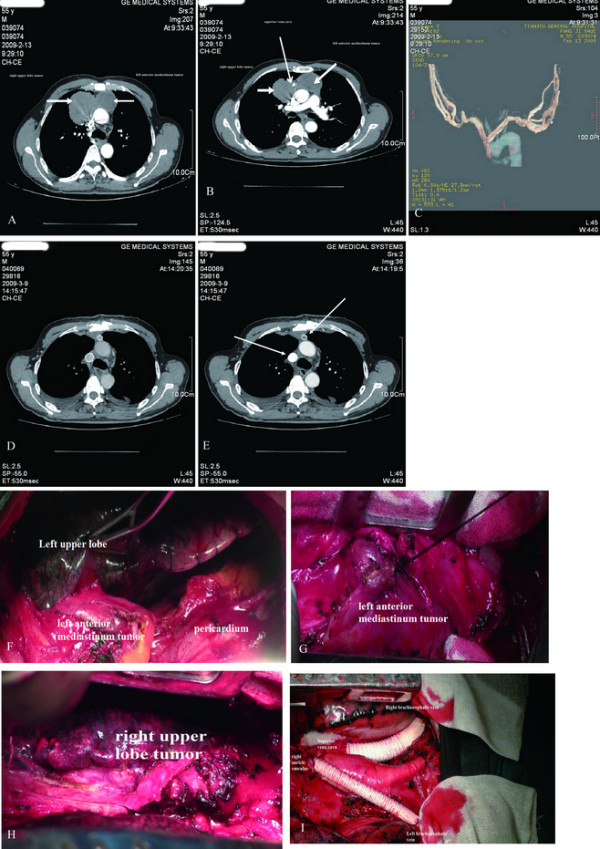
**Chest computed tomographic scan**. Figure 1A, 1B An enhanced Chest computed tomographic scan revealed a mass in the anterior segment of the right upper lobe with continuation to some mediastinal swelling lymph nodes. Multiple swelling lymph nodes could be found in the mediastinum. Figure C Three-D reconstruction showed the superior vena cava, whose lumen was unobstructed but deformated under the compression of the mass. Figure 1D, 1E Postoperative enhanced Chest computed tomographic scan images. Figure 1F, 1G Surgical findings of the mediastinal mass. Figure 1H Surgical findings of the intrapulmonary mass. Figure 1I Reconstruction of left brachiocephalic vein to right auricle and reconstruction of right brachiocephalic vein to superior vena cava.

**Figure 2:**
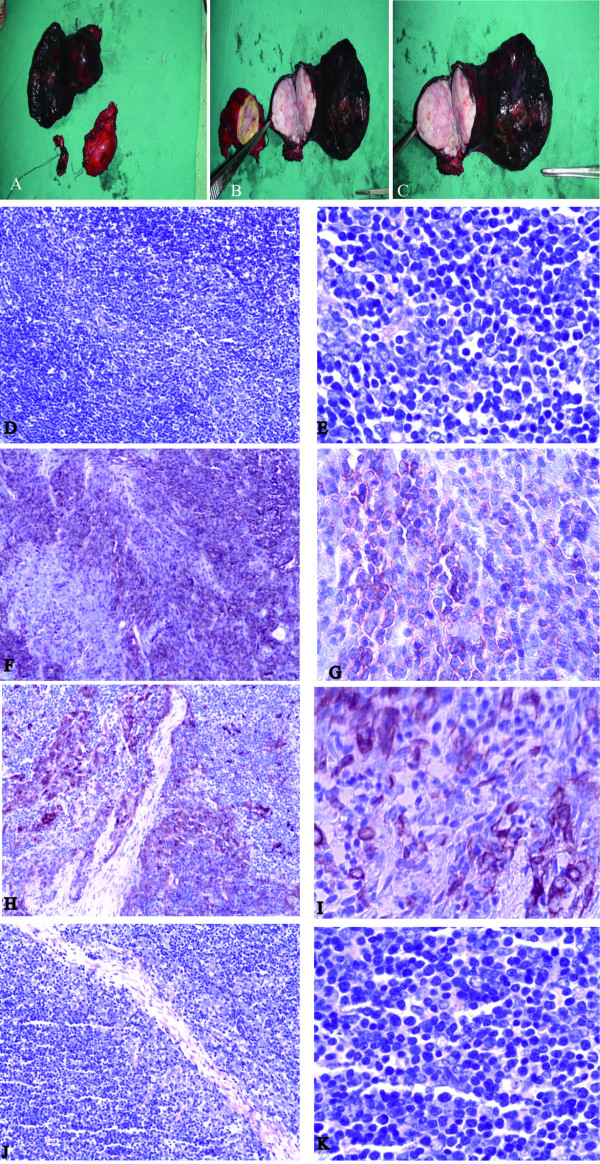
**Macro and microscopic observations**. Figure 2A,2B,2C The resected intrapulmonary and mediastinal tumors(3.5 cm × 4.0 cm × 5 cm and 6 cm × 6 cm × 6 cm, respectively). Figure 2D Histological findings of the primary intrapulmonary tumor(PIT), H&E × 100. Figure 2E PIT H&E × 400. Figure 2F PIT CD99 Immunohistochemistry × 100. Figure 2G PIT CD99 Immunohistochemistry × 400. Figure 2H PIT CK 5& CK6 Immunohistochemistry × 100. Figure 2I PIT CK 5& CK6 Immunohistochemistry × 400. Figure 2J Histological findings of the primary mediastinal tumor(MT), H&E × 100. Figure 2K MT, H&E × 400.

A 55-year-old Chinese man was admitted with a history of progressive exertional dyspnea of 55 days' duration and a radiological finding of an anterior mediastinal mass for 7 days. The patient had no clinical features of myasthenia gravis. An enhanced Chest computed tomographic scan revealed a 5.5 cm × 6.0 cm × 4.1 cm mass in the anterior segment of the right upper lobe with continuation to some mediastinal swelling lymph nodes. Multiple swelling lymph nodes could be found in the mediastinum (Figure 1A, B, Figure 2A, B, C). Three-D reconstruction showed the superior vena cava, whose lumen was unobstructed but deformated under the compression of the mass (Figure 1C). A computed tomographic scanning of the brain and bones were normal. An exploratory limited right thoracotomy was undertaken through a median sternotomy. A soft encapsulated mass(3.5 cm × 4.0 cm × 5 cm) was found in the left anterior superior mediastinum, with invasion to the left pericardium and visceral pleura, adhesive to partial superior lobe of right lung and brachiocephalic vein(Figure 1G, I). In the anterior segment of the right upper lobe, a mass was 6 cm in diameter, invading the junction of right and left brachiocephalic veins and upper segment of superior vena cava (Figure 1F, H). Both masses are solitary. Therefore resection of the mass in the left anterior superior mediastinum and upper lobectomy of right lung were performed, with lymph Nodes clearance, superior vena cava, left and right brachiocephalic veins resection, reconstruction of left brachiocephalic vein to right auricle and reconstruction of right brachiocephalic vein to superior vena cava. Microscopically according to the WHO classification, the mediastinal tumor(MT) was a B3/B2 primary thymoma and the mass in the upper lobe of right lung is mainly a B3/B2 primary intrapulmonary thymoma(PIT) with local A type tumors. Histologic evaluation indicated that, CK5 & CK6 +, EMA + locally, CD5 -, CD99 +(Figure 2. No lymph metastasis was found. Warfarin was applied to the patient as anticoagulation and 50 Gy mediastinal irradiation was given as adjuvant therapy. The patient has since recovered uneventfully and is now being followed up as an outpatient (Figure 1D, E). After follow-up of eight months, there was no significant metastasis or recurrence found by radiological examinations.

## Discussion

Primary intrapulmonary thymuses are very uncommon, with 28 cases reported to date[2]. Even rarer cases were reported for Synchronous primary intrapulmonary and mediastinal thymoma. The incidence for lung cancer in China increased by 1.63% from 1988 to 2005. Some special thoracic malignancies should be paid attention to in China[3]. Primary intrapulmonary thymomas appear to fall into two groups: one is in the hilus of the lung, in relation to the wall of a major bronchus or attached to the pericardium, and the other is peripheral in the lung and beneath the visceral pleura[4]. In the case of hilar type, the notion that intrapulmonary thymomas are derived from mediastinal thymomas that migrate into the lung with pinching off of the pleural behind them would be acceptable, but it fails to provide a satisfactory explanation for the occurrence of the peripheral[2]. Marchevsky[5] believed intrapulmonary thymomas arose from stem cells, uncommitted germinative cells capable of differentiating along a variety of lines. This theory is supported by the large number of reports of heterotopic, histologically mature tissues within the lung parenchyma, such as thyroid follicules, pancreas, adrenal, liver, neuro-glial tissue and endometrium, or tumors derived from ectopic tissue, such as melanoma, meningioma, glomus or glomangioma, choriocarcinoma, teratoma, ependymoma and, of course, thymoma, which could develop from such stem cells. Multiple thymomas remain controversial as to whether multiple thymomas involve intrathymic dissemination or represent multiple primaries, which could be explained by Marchevsky's theory. Although Bernatz et al. [6] reported 3 out of 138 (2.2%) thymomas to be multiple primaries, it was difficult to clarify whether the multiple thymomas in their cases involved double primary or dissemination, because they did not mention any close histological characteristics among the multiple thymomas. Since both our cases were totally encapsulated tumors and did not have any dissemination in the other portion, they were considered to be multiple primaries[7]. Therefore our case provided better evidence to support Marchevsky's theory for the development of intrapulmonary thymomas.

The clinical course is that of a slow-growing lesion that remains asymptomatic until it reaches a size causing problems due to local growth, such as pain, bronchial obstruction or hemoptysis. As with mediastinal thymomas, they can be associated with paraneoplastic syndromes, such as myasthenia gravis or Good's syndrome. Resection appears sufficient in non-malignant tumors. In incompletely resected patients, adjuvant radiotherapy should be considered. Long-term regular clinical follow-up is warranted, because of the risk of late local recurrence.

## Consent

Written informed consent was obtained from the patient for publication of this case report and any accompanying images. A copy of the written consent is available for review by the Editor-in-Chief of this journal.

## Competing interests

The authors declare that they have no competing interests.

## Authors' contributions

ZS, JC and QZ were the primary caregiver for this patient and reviewed the manuscript. SL and SW also cared for this patient. XX performed data collection and drafted the manuscript. All authors read and approved the final manuscript.

## References

[B1] Engels EA Pfeiffer RM Malignant thymoma in the United States: demographic patterns in incidence and associations with subsequent malignancies Int J Cancer 2003 105 546 551 10.1002/ijc.11099 12712448

[B2] Ishibashi H Takahashi S Tomoko H Shibuya J Suzuki S Handa M Primary intrapulmonary thymoma successfully resected with vascular reconstruction Ann Thorac Surg 2003 76 1735 1737 10.1016/S0003-4975(03)00654-4 14602330

[B3] Chen WQ Zhang SW Zou XN Evaluation on the incidence, mortality and tendency of lung cancer in China Thoracic Cancer 2010 1 35 40 10.1111/j.1759-7714.2010.00011.x 27755783

[B4] Kalish PE Primary intrapulmonary thymoma N Y State J Med 1963 63 1705 1708 13961941

[B5] Marchevsky AM Lung tumors derived from ectopic tissues Semin Diagn Pathol 1995 12 172 184 7638449

[B6] Bernatz PE Harrison EG Clagett OT Thymoma: a clinicopathologic study J Thorac Cardiovasc Surg 1961 42 424 444 13868094

[B7] Okada M Tsubota N Yoshimura M Miyamoto Y Sakamoto T Two cases of synchronous multiple thymoma Surg Today 1998 28 1323 1325 10.1007/BF02482827 9872561

